# IMPACT OF A SIMULATED LAPAROSCOPIC TRAINING PROGRAM IN A THREE-YEAR GENERAL SURGERY RESIDENCY

**DOI:** 10.1590/0102-672020190001e1436

**Published:** 2019-04-29

**Authors:** Rodrigo TEJOS, Rubén AVILA, Martin INZUNZA, Pablo ACHURRA, Richard CASTILLO, Anne ROSBERG, Octavio CORDERO, Rodrigo KUSANOVICH, Felipe BELLOLIO, Julián VARAS, Jorge MARTÍNEZ

**Affiliations:** 1 Center of Experimental Surgery and Simulation, Department of Digestive Surgery, School of Medicine, Pontificia Universidad Católica de Chile, Santiago, Chile;; 2 Department of Digestive Surgery, School of Medicine, Pontificia Universidad Católica de Chile, Santiago, Chile; 3 International Internship, School of Medicine, Albert-Ludwigs-University of Freiburg, Baden-Württemberg, Germany.

**Keywords:** Medical education, General surgery, Patient simulation, Educação médica, Cirurgia geral, Simulação, Paciente

## Abstract

**Background:**

A General Surgery Residency may last between 2-6 years, depending on the country. A shorter General Surgery Residency must optimize residents’ surgical exposure. Simulated surgical training is known to shorten the learning curves, but information related to how it affects a General Surgery Residency regarding clinical exposure is scarce.

**Aim:**

To analyze the effect of introducing a validated laparoscopic simulated training program in abdominal procedures performed by residents in a three-year General Surgery Residency program.

**Methods:**

A non-concurrent cohort study was designed. Four-generations (2012-2015) of graduated surgeons were included. Only abdominal procedures in which the graduated surgeons were the primary surgeon were described and analyzed. The control group was of graduated surgeons from 2012 without the laparoscopic simulated training program. Surgical procedures per program year, surgical technique, emergency/elective intervention and hospital-site (main/community hospitals) were described.

**Results:**

Interventions of 28 graduated surgeons were analyzed (control group=5; laparoscopic simulated training program=23). Graduated surgeons performed a mean of 372 abdominal procedures, with a higher mean number of medium-to-complex procedures in laparoscopic simulated training program group (48 vs. 30, p=0.02). Graduated surgeons trained with laparoscopic simulated training program performed a higher number of total abdominal procedures (384 vs. 319, p=0.04) and laparoscopic procedures (183 vs. 148, p<0.05).

**Conclusions:**

The introduction of laparoscopic simulated training program may increase the number and complexity of total and laparoscopic procedures in a three-year General Surgery Residency.

## RESUMO

**Racional:**

Residência em Cirurgia Geral pode durar entre 2-6 anos, dependendo do país. Residência mais curta deve otimizar a exposição dos residentes às cirurgias. Sabe-se que o treinamento cirúrgico simulado encurta as curvas de aprendizado, mas a informação relacionada à como isso afeta a residência em relação à exposição clínica é escassa.

**Objetivo:**

Analisar o efeito da introdução de um programa de treinamento laparoscópico simulado validado em procedimentos abdominais realizados por residentes em um programa de Residência em Cirurgia Geral de três anos.

**Métodos:**

Um estudo de coorte não simultâneo foi desenhado. Quatro gerações (2012-2015) de cirurgiões graduados foram incluídos. Apenas os procedimentos abdominais em que os cirurgiões graduados foram o cirurgião principal foram descritos e analisados. O grupo controle foi de cirurgiões graduados de 2012 sem programa de treinamento laparoscópico simulado. Procedimentos cirúrgicos por ano de programa, técnica cirúrgica, intervenção de emergência ou eletiva e local do hospital (hospitais principais/comunitários) foram descritos.

**Resultados:**

Intervenções de 28 cirurgiões graduados foram analisadas (controle=5; programa de treinamento simulado=23). Os cirurgiões graduados realizaram média de 372 procedimentos abdominais, com maior número médio de procedimentos de médio a complexo no grupo de programa de treinamento simulado (48 vs. 30, p=0,02). Cirurgiões graduados treinados com programa de treinamento simulado realizaram número maior de procedimentos abdominais totais (384 vs. 319, p=0,04) e procedimentos laparoscópicos (183 vs. 148, p<0,05).

**Conclusões:**

A introdução do programa de treinamento laparoscópico simulado pode aumentar o número e a complexidade dos procedimentos totais e laparoscópicos na Residência em Cirurgia Geral de três anos.

## INTRODUCTION

The aim of a General Surgery Residency (GSR) is to attain an autonomous and competent specialist through training in technical and non-technical skills^13^ ^,^ ^27^. Structured programs with specific objectives are necessary to ensure the acquisition of the required skills^13^. The duration of GSR is usually defined by regulatory institutions in each country, considering socioeconomic realities, sanitary and epidemiological changes. Worldwide, the duration has been established from two to six years^14,15,19, 23,25^. In our country, the Chilean Society of Surgeons together with the Ministry of Health has defined a three-year training period for the General Surgery Residency^17^ ^,^ ^19^. 

Initially, the mentorship model proposed by William Halsted (1889)^21^ was used as the teaching method for all surgical techniques. However, surgery and surgical education have evolved since Halsted. Legislation regarding residents’ working hours; a greater number of residents per year; high institutional costs involved in student formation; progress in minimally invasive surgery; along with the need to provide safe care to the patient, have all led to development of novel educational methods to complement the traditional model^2^ ^,^ ^3^ ^,^ ^6^. Simulation training programs have shortened learning curves even for complex techniques such as advanced laparoscopy, using a safe and efficient environment where deliberate practice and effective feedback is applied^2^ ^,^ ^10^ ^,^ ^24^. Simulation has been progressively introduced into GSR around the world with training programs as FLS^30^. Since 2010 the Pontificia Universidad Católica de Chile’s (PUC) GSR program incorporated a validated laparoscopic simulated training program (LSTP), becoming the first Latin-American University to include this educational method into its formal curriculum^7^ ^,^ ^8^ ^,^ ^9^ ^,^ ^29^. Although this learning method proves effective to acquire and improve laparoscopic surgery skills, there are no studies that analyze the effect of this intervention in the quantity and type of laparoscopic procedures performed by the residents of an intensive three-year surgery program.

This article aims to describe the outcomes of a three-year GSR program and to analyze the effect of introducing a validated LSTP in the amount and complexity of abdominal surgical procedures performed by the trainees during their residency.

## METHODS

### Population and data collection

We designed a non-concurrent cohort study and invited all GS graduated between the years 2012-2015 (four generations) from the PUC’s GSR program to participate. Residents prospectively registered all surgical procedures during their GSR, and this data was gathered and evaluated for results. Only abdominal surgical interventions (open and laparoscopic approaches) in which the resident was the primary surgeon were described and analyzed. 

The PUC’s GSR program has four hospitals associated with our institution, two of these being community hospitals outside of Santiago city. 

To assess the effects on the amount and complexity of abdominal procedures performed by residents after the incorporation of a simulated training program, we defined two groups. GS graduated in 2012 were identified as the control group. They were trained exclusively using the traditional method, without the laparoscopic simulation-training program (NLSTP). The intervention group consisted of GS graduated in 2013, 2014 and 2015; who were trained with the validated LSTP^7^ ^,^ ^8^ ^,^ ^9^ ^,^ ^29^.

Surgical procedures per program year (PGY); surgical technique (open or laparoscopic); priority of the intervention (emergency or elective); and hospital-site (community hospitals versus our main tertiary hospitals) were described.

We calculated an annual-surgical-exposure index (ASE) and an annual resident-per-attending-surgeon index (RPS) to analyze the effects of institutional changes on residents’ surgical exposition in the follow-up period.

### Interventions 

#### General Surgery Residency Program description

In Chile, surgical training is completed in two stages. The first stage is a three-year GSR program, followed by a fellowship which lasts two to three years. The possibility of performing this second step depends on the resident’s curricular excellence, thus not all GS obtain a fellowship. Additionally, formal research fellowships may be undertaken and complement any of these two stages.

The GSR in our medical school is a three-year structured training program. It includes rotations through different specialty teams and Departments, of which upper GI and Bariatric, Hepato-biliary-pancreatic, Colorectal surgery and Emergency Departments are the rotations where trainees may assume abdominal procedures. All rotations are designed in a step-by-step scheme to obtain knowledge of progressively increasing complexity and responsibility to attain, by the end of PGY3, mastery in surgical problem-solving. 

Resident training is done using a mentorship system with attending surgeons; clinical case discussions with decision-making; and the direct assessment and teaching of surgical techniques. Finally, since 2010 the LSTP was incorporated into the GSR curriculum program. 

### Laparoscopic simulated training program (LSTP) 

Currently, simulated training is mandatory for our general surgery residents, and it contributes to the acquisition of laparoscopic skills. This training consists of two validated courses for acquiring basic and advanced laparoscopic dexterity^7^ ^,^ ^8^ ^,^ ^9^ ^,^ ^29^. Our research group has demonstrated that the skills obtained with the advanced LSTP transfer adequately to the operating room^7^. Residents must complete these courses in PGY1 of the GSR program; otherwise, they cannot continue to PGY2.

**FIGURE 1:**
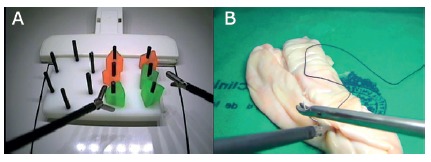
A) Basic Laparoscopic Simulation Program (LSTP); B) advanced LSTP

The basic LSTP is a competency-based program composed of FLS and virtual reality exercises, lasting between 25 to 50 hours^28^ ^,^ ^30^. It aims to develop basic laparoscopic skills using progressively exercises in synthetic simulated models. The course has a practical and a theoretical component. The practical module consists of 12 sequential complexity stations of simulated training (Figure 1A). 

The advanced LSTP lasts 40 to 50 hours and aims to develop advanced laparoscopic skills through progressively difficult exercises in simulated models with ex-vivo tissue. At the end of the program, students must perform a laparoscopic jejuno-jejunostomy in less than 20 minutes, which is considered as a complex laparoscopic procedure. Like the basic LSTP, the advanced course has a practical and a theoretical module (Figure 1B). A central component of both basic and advanced LSTP is the effective feedback given to the residents. With this educational tool, we clarify learning objectives, reinforce positive aspects and provide the basis for correcting errors through deliberate practice^16^ ^,^ ^20^.

### Educational environment changes analysis

To analyze the effect of institutional changes on residents’ surgical exposition in the follow-up period, we described the number of abdominal procedures per year; the number of attending surgeons per year; and the number of residents per year in the follow-up period. We calculated an annual-surgical-exposure index (ASE) defined as the proportion of abdominal procedures per year divided by the total number of residents per year (PGY 1, 2 and 3). Furthermore, we calculated a resident-per-attending-surgeon index (RPS) defined as the proportion of attending surgeons per year divided by the total number of residents per year (PGY1, 2 and 3).

### Statistical analysis

Kolmogorov Smirnov test was performed to evaluate the normality of the data. The continuous variables were analyzed with Student’s T-test and expressed as mean and range. Chi-squared test was used to compare categorical variables and are summarized as the number of cases (n) and percentage of the total. ANOVA test for related samples was used to compare the ASE and RPS index. A value of p<0.05 was considered as statistically significant. All descriptive and statistical analysis was performed using SPSS Statistics ® 22, 0 (Chicago, USA).

## RESULTS

### Abdominal surgical procedures in a 3-year GSR

Records of 28 out of 34 (82%) residents graduated between 2012 and 2015 (four generations) were included. The control group (NLSTP) comprised 5 GS graduated in 2012. The LSTP group included 23 GS graduated from 2013 to 2015. A total of 10,415 abdominal interventions were performed by the 28 trainees as primary surgeons, always under direct supervision. There is a progression in the number of procedures performed as the primary surgeon concerning to the PGY of residency, with a total of 1,702 (22.1%), 2,788 (36.3%) and 3,198 (41.6%) for PGY1, PGY2, and PGY3 respectively (Table 1). 

**TABLE 1:** Abdominal procedures in a 3-year general surgery residency

Surgery	PGY1 n= 2302 (22.1%)	PGY2 n= 3781 (36.3%)	PGY3 n=4332 (41.6%)	Total n=10415 (100%)	Mean number per resident
Laparoscopic cholecystectomy^†^	1056 (45.9%)	1062 (28.1%)	1163 (26.8%)	3281 (31.5%)	117
Classic appendectomy^†^	573 (24.9%)	1034 (27.4%)	949 (21.9%)	2556 (24.54)	91
Open hernia repair surgery^†^	348 (15.1%)	522 (13.8%)	359 (8.3%)	1229 (11.8)	44
Laparoscopic appendectomy^†^	37 (1.6%)	136 (3.6%)	793 (18.3%)	966 (9.28)	35
Classic cholecystectomy^†^	143 (6.2%)	268 (7.1%)	290 (6.7%)	701 (6.73)	25
Exploratory laparotomy^†^	44 (1.9%)	147 (3.9%)	221 (5.1%)	412 (3.96)	15
Colorectal resections ^† *^	14 (0.6%)	132 (3.5%)	178 (4.1%)	324 (3.1)	12
Gastroenteroanastomosis, gastrostomy or perforated peptic ulcer^† *^	44 (1.9%)	91 (2.4%)	121 (2.8%)	256 (2.5)	9
Hepatobiliary surgery^† *^	18 (0.8%)	110 (2.9%)	108 (2.5%)	236 (2.3)	8
Small bowel resection^† *^	2 (0.1%)	163 (4.3%)	3 (0.06%)	168 (1.6)	6
Transit reconstitution^† *^	23 (1%)	72 (1.9%)	61 (1.4%)	156 (1.5)	6
Gastrectomy (partial or total) ^† *^	-	34 (0.9%)	65 (1.5%)	99 (0.9)	4
Splenectomy or pancreatectomy^† *^	-	8 (0.2%)	17 (0.4%)	25 (0.2)	1
Gastroesophageal reflux surgery^† *^	-	2 (0.04%)	1 (0.03%)	3 (0.03)	0.1
Organ procurement surgery^† *^	-	-	3 (0.06%)	3 (0.03)	0.1
Total^†^	2302 (100%)	3781 (100%)	4332 (100%)	10415 (100%)	372

^†^=Number of cases/percent; *=intermediate - complex procedures

Laparoscopic cholecystectomy, classic appendectomy, and open hernia repair surgery were the most frequent, accounting for 67.8% of all surgical interventions, with a mean number of procedures performed by each resident of 117 (66-176), 91 (54-142), and 44 (23-71), respectively. Laparoscopic cholecystectomy was the most frequent procedure in all years, and laparoscopic appendectomy was mainly performed in the last year of the program. More complex surgeries such as colorectal resections, hepatobiliary procedures, gastrectomy, splenectomy, and pancreatectomy were less frequent, accounting for only 12.2% of all surgical interventions (Table 1). All residents spent an average of four months (equivalent to a 12% of the complete program) at the community hospitals between PGY2 and PGY3, where they performed 29.7% of all the residency procedures. Most procedures were performed in a tertiary hospital and in an emergency setting with no statistically differences between groups. (Table 2).

**TABLE 2:** Procedures characteristics

Variable	Total	NLSTP n=5	LSTP n=23	p-value
Procedimento‡				NS
Elective	4253 (40,8%)	654 (41%)	3599 (40,8%)	
Emergency	6162 (59,2%)	940 (59%)	5222 (59,2%)	
Hospital’s complexity level^‡^				NS
Tertiary	7324 (70,3%)	1140 (71,5%)	6184 (70,1%)	
Community hospitals	3091 (29,7%)	454 (28,5%)	2637 (29,9%)	

### Impact of a laparoscopic simulated training program

**FIGURE 2:**
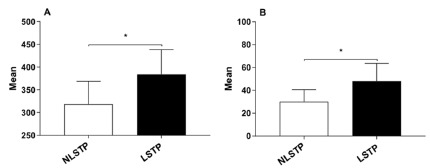
A) Mean and standard deviation of all abdominal procedures; B) intermediate-complex procedures during a 3-year surgery

The mean number of total abdominal procedures and intermediate-complex abdominal procedures (defined in Table 1) performed by LSTP group was statistically superior to NLSTP group, [384 (272-474) vs 319 (260-381) (p=0.04)] and [48 (30-55) vs 30 (21-43) (p=0.02)] respectively (Figures 2A, 2B). 

(*)=Statistically significant difference (p ˂ 0.05); NS=non-significant difference

**FIGURE 3:**
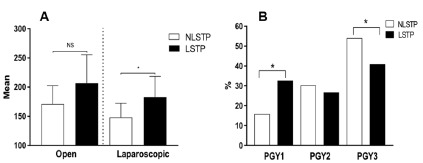
A) Mean and standard deviation of laparoscopic and open surgical technique during a 3-year surgery residence; B) distribution of laparoscopic procedures per year of residence

The analysis of laparoscopic procedures is shown in Figure 3. The LSTP group performed a statistically superior mean number of laparoscopic procedures to the NLSTP group [183 (129-240) vs 148 (118-176) (p˂ 0.05)] without statistically differences in the number of open procedures [207 (114-290) vs 171 (133-218) (p>0,05) respectively] (Figure 3A). Furthermore, considering the distribution of laparoscopic procedures per year of residency, the LSTP group performed a higher percentage of laparoscopic procedures in their first year of residency (PGY1) [32.6% vs 15.8% (p˂ 0.05)] whereas the NLSTP group performed the majority of laparoscopic procedures in PGY3 (54%, Figure 3B).

(*)=Statistically significant difference (p ˂ 0.05); NS=non-significant difference

### Educational environment changes analysis

**FIGURE 4:**
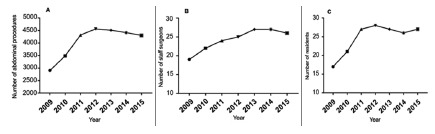
A) Number of abdominal procedures performed per year; B) number of attending surgeons per year; C) number of residents per year

A progressive increase was observed in the number of annual abdominal procedures, attending surgeons and residents per year during the follow-up period (Figure 4).

**FIGURE 5:**
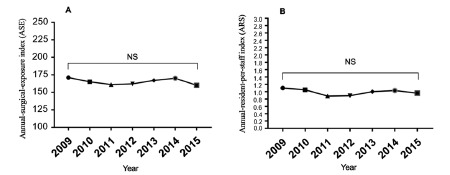
A) Annual-surgical-exposure index (ASE) calculated as number of abdominal procedures divided into number of residents per year; B) annual resident-per-attending-surgeon index (RPS) calculated as number of attending surgeons divided into number of residents per year

Nevertheless, when we calculated the ASE and RPS index, an average exposure of 165 surgical procedures per year for each resident and an attending surgeon/resident ratio of 1:1 was observed without significant differences in the follow-up period (Figures 5A, 5B).

(*)=Statistically significant difference (p ˂ 0.05); NS=non-significant difference

## DISCUSSION

New technological developments, increased specialization, and high-quality requirements for safety standards in patient care, has led to most GSR to adapt their surgical learning methods^2^ ^,^ ^24^. Laparoscopy for general surgeons was initially introduced mainly for basic procedures, but with time its application has been extended to more complex scenarios. In the early stages, teaching laparoscopic skills in general surgery residencies was tough and not standardized, considering that it demands developing motor and spatial skills which are more difficult to acquire^3^ ^,^ ^21^ ^,^ ^22^. To deal with these challenges, our institution has incorporated changes in the GSR considering the following elements: teaching using learning by practice under supervision; step-by-step training with increasing complexity; a closer teaching environment; generous tutoring by surgeon teachers; and, more recently, the LSTP.

We observed a significant increase in the number of abdominal procedures after the incorporation of a simulated training program early in our GSR. Although with this type of study it is not possible to demonstrate a causal relationship, we consider it a proper approach due to ethical limitations of leaving a group of residents without simulation training. Many authors have postulated that simulated training increases the number of laparoscopic clinical practice, which could support these findings^5^ ^,^ ^11^ ^,^ ^12^. The increasing number of laparoscopic procedures from PGY1 to PGY3 (Figure 3C) and the progressive rise in complex laparoscopic procedures as primary surgeon coincide with the formal introduction of simulated training into the GSR curriculum. Simulation has played a crucial role in the acquisition of surgical skills; transfer of these skills to the operating room; and shortening of learning curves in the acquirement of laparoscopic skills^1^ ^,^ ^4^ ^,^ ^29^. The LSTP has allowed first-year residents at early stages to develop know-how in laparoscopy, by delivering tools that may facilitate their access to more surgical cases. Additionally, simulation enables trained PGY1 residents to perform common surgeries such as laparoscopic appendectomy and cholecystectomy. Combined with clinical experience this lays down the foundation for competency in laparoscopy, possibly allowing residents to address more complex procedures^21^ ^,^ ^22^. When asking expert tutor surgeons, they notice greater confidence in their trained junior residents and hence, allow them to perform more surgeries at early stages. 

Laparoscopic cholecystectomy is the most frequently performed abdominal intervention by residents, which is expected given the high incidence of cholelithiasis in our country^18^. This high number of laparoscopic cholecystectomies wields an essential part during the process of learning and acquisition of laparoscopic skills of our residents. 

The quantity of laparoscopic interventions performed over a period of three years by PUC’s GS residents is notable and reflects the aims set in the design of the program. These results meet the requirements of actual surgery; reinforce the concepts of teaching competencies and learning-by-practice; and emphasize training with new technologies with a gradual increase in responsibility and complexity of the assigned tasks. Having diverse clinical institutions, including the ones located in community hospitals, seems to allow a more integral training of residents. Residents’ rotations at community hospitals outside of our capital Santiago has been an enriching element for the GSR at PUC, allowing trainees to perform a higher number of open procedures in emergency settings. Community hospitals benefit from these rotations as residents not only perform many surgical procedures but also push these institutions to obtain new technologies such as laparoscopic instrumentation. The percentage of all abdominal procedures performed in community hospitals were 29.7%. Interestingly, residents only rotate in these hospitals during a four-month period (equivalent to a 12% of their three-year program). This finding confirms the importance of these types of rotations as part of the GSR curriculum.

The main limitation of the study lies in the higher number of GS residents trained under LSTP compared to the control group (NLSTP), with a 3:1 ratio. This occurred due to a combination of two factors: the prospective registry of surgical procedures began in 2009 and LSTP became mandatory for all incoming generations of GS residents from 2010. Therefore, only one generation without LSTP had available records. Despite this limitation, it is statistically permissible^26^.

Educational environment changes analysis was performed to assess possible factors that could influence the number of surgeries undertaken per resident. During the study period, we observed an increase in the number of annual surgical procedures and in the number of attending surgeons and residents per year. Nevertheless, the steadiness of the ASE and RPS indices could explain that the increase in both total and laparoscopic abdominal surgeries performed by residents were not affected by changes in these variables, but by the introduction of the LSTP. 

## CONCLUSION

General surgeons graduated from a three-year residency program performed diverse abdominal procedures in each PGY. The incorporation of a laparoscopic simulated training program appears to increase the amount and complexity of total abdominal procedures and laparoscopic procedures performed by the trainees during their residency.
